# Patient-Reported Visual Function and Vision-Related Quality of Life Across Ophthalmic Diseases: An Exploratory Cross-Sectional Study

**DOI:** 10.3390/jcm15156128

**Published:** 2026-08-06

**Authors:** Kenji Kashiwagi, Syowta Fukuba, Hiroshi Kasai, Yoshiko Fukuda, Shuhei Hosoda, Naohiko Tanabe

**Affiliations:** 1 Department of Ophthalmology, Interdisciplinary Graduate School of Medicine, University of Yamanashi, 1110 Shimokato, Chuo City 409-3898, Japan; hkasai@yamanashi.ac.jp (H.K.); ysugiyama@yamanashi.ac.jp (Y.F.); hosodas@yamanashi.ac.jp (S.H.); tanabe@yamanashi.ac.jp (N.T.); 2 Bibai Suzuran Clinic, Bibai City 072-0011, Japan; syowta_fukuba@kaze-suzuran.com; 3 Tanabe Ophthalmic Clinic, Kai City 400-0117, Japan

**Keywords:** patient-reported outcomes, vision-related quality of life, VFQ-25, subjective visual function, questionnaire, ophthalmology, visual acuity

## Abstract

**Background/Objectives:** Patient-reported outcomes (PROs) provide information on subjective visual function that is not fully captured by conventional clinical measures. This exploratory cross-sectional study evaluated item-level responses to an original 11-item questionnaire and their association with vision-related quality of life across ophthalmic diseases. **Methods:** A total of 262 participants, including patients with glaucoma, age-related macular degeneration, diabetic retinopathy, retinitis pigmentosa, cataract, and individuals with long-standing severe visual impairment, completed the questionnaire and the National Eye Institute Visual Function Questionnaire-25 (VFQ-25). Associations with age, disease category, and better-eye visual acuity were examined using correlation analyses, multivariable regression, and ordinal logistic models. **Results:** Perceived change in vision after treatment or disease management (A6) showed the most consistent item-level associations. A6 remained associated with age after adjustment for disease category and visual acuity (β = −0.030, *p* < 0.001), whereas visual acuity was not associated with questionnaire domains. A6 was negatively correlated with the VFQ-25 composite score (r = −0.693, *p* < 0.001) and remained associated with this score in multivariable analysis (β = −0.230, *p* < 0.001). **Conclusions:** Item-level PRO responses varied across clinical backgrounds and were not adequately explained by visual acuity alone. These findings suggest that patient-perceived visual change after treatment or disease management may provide complementary information on vision-related quality of life. Further studies using validated instruments and disease-specific severity measures are needed.

## 1. Introduction

Patient-reported outcomes (PROs) have become increasingly important in ophthalmology because they capture subjective aspects of visual function, symptoms, treatment experience, and quality of life that are not fully reflected by conventional clinical measures such as visual acuity [1,2,3,4,5,6,7,8,9,10,11]. Visual acuity remains a standard clinical indicator, but it does not necessarily reflect patients’ lived experiences, including functional limitations, psychological impact, adaptation to visual impairment, and treatment-related perceptions.

PROs are influenced by multiple factors, including age, disease characteristics, treatment history, and broader psychosocial context [8,12,13,14]. In ophthalmology, disease-specific features such as visual field impairment, binocular visual function, contrast sensitivity, and disease severity may also contribute to patient-reported outcomes. However, these variables are not always uniformly available across different ophthalmic diseases in routine clinical settings.

Previous studies have shown that discrepancies may exist between objective clinical measures and subjective patient experience [15,16,17]. Many studies have focused on composite quality-of-life measures, which are useful for summarizing overall vision-related burden but may obscure item-level differences in patient perception. Therefore, exploratory item-level analyses may help identify which patient-reported domains are most closely related to clinical background factors and vision-related quality of life.

Although numerous validated generic, vision-specific, and disease-specific PRO instruments are available, comprehensive multi-item questionnaires may be difficult to incorporate into routine outpatient care because of the time and effort required for completion and scoring. Conversely, a brief questionnaire applicable across heterogeneous ophthalmic conditions may reduce respondent burden but may overlook experiences that are particularly relevant to specific diseases. Recent studies have shown that the functional and emotional effects of eye disease vary according to disease type and severity, supporting the need for disease-sensitive or disease-tailored assessment [5,11,18]. Therefore, there remains value in exploring concise item-level domains that can be administered with minimal respondent burden and subsequently refined for disease-specific applications.

Against this background, we used an investigator-developed 11-item exploratory questionnaire to assess subjective visual function, psychological impact, treatment-related perceptions, and disease perception. The questionnaire was not intended to replace established validated instruments, serve as a universally applicable ophthalmic PRO measure, or provide a formal assessment of psychiatric status. Rather, the item-level analysis was intended to identify candidate domains showing potentially disease-sensitive response patterns while minimizing respondent burden. We also administered the National Eye Institute Visual Function Questionnaire-25 (VFQ-25) to examine the associations between these exploratory item-level responses and an established measure of vision-related quality of life. Specifically, this study evaluated the associations of individual questionnaire items with age, disease category, better-eye visual acuity, surgical history, and VFQ-25 composite score. Because the original questionnaire has not been formally validated as an independent instrument, all analyses were considered exploratory and hypothesis-generating.

## 2. Materials and Methods

Declaration of Generative AI and AI-Assisted Technologies: During the preparation of this manuscript, the authors used ChatGPT-5.5 (OpenAI, San Francisco, CA, USA) solely for English-language editing, grammar correction, and improvement of readability. ChatGPT was not used for data analysis, statistical analysis, interpretation of results, generation of scientific conclusions, or creation of figures. The authors reviewed and edited all AI-assisted outputs and take full responsibility for the final content of the manuscript.

### 2.1. Study Design and Ethical Approval

This exploratory cross-sectional study was conducted between June and September 2025. The study adhered to the tenets of the Declaration of Helsinki. Written informed consent was obtained from all participants. The study protocol was approved by the Ethics Committee of the University of Yamanashi School of Medicine (Approval Code: 2864).

### 2.2. Participants

The disease-specific groups included patients with glaucoma, age-related macular degeneration (AMD), diabetic retinopathy (DMR), retinitis pigmentosa (RP), and cataract. These groups consisted of consecutive patients who met the inclusion and exclusion criteria during the study period, were regularly receiving treatment at the University of Yamanashi Hospital or Tanabe Eye Clinic, and provided informed consent. Cataract was included as a common, potentially reversible condition to provide a clinically informative contrast with chronic, generally irreversible retinal and optic nerve diseases. Differences in prognosis and treatment effectiveness were considered when interpreting between-disease comparisons. All of the AMD patients enrolled in this study had neovascular AMD. Patients with multiple coexisting ophthalmic diseases that could not be clearly assigned to a single primary diagnosis were excluded from disease-specific analyses. Patients with known psychiatric disorders or dementia, identified through medical history interviews, were also excluded.

During the same study period, individuals from a visually impaired support group, Yuimal, were also included to broaden the range of visual function and patient-reported experiences. This group consisted of individuals with long-standing severe visual impairment who were not receiving active ophthalmic treatment at the time of the study. Because the underlying ocular diseases were heterogeneous and were not restricted to a single diagnostic category, this group was interpreted descriptively and was also excluded in sensitivity analyses.

### 2.3. Clinical Data

Clinical data were collected from medical records at the time of questionnaire administration. The following variables were obtained: age, sex, disease category, surgical history, and visual acuity. Visual acuity was measured using a standard decimal visual acuity chart under routine clinical conditions and converted to logarithm of the minimum angle of resolution (logMAR) units for statistical analysis. Better-eye visual acuity was used in the analyses.

### 2.4. Original 11-Item Patient-Reported Questionnaire

Patient-reported outcomes were assessed using a structured questionnaire consisting of 11 items (A1–A11), covering subjective visual function, psychological impact, treatment-related perceptions, and disease perception (Table 1). The questionnaire was developed by the study investigators for this exploratory study through discussion among the participating ophthalmologists; it was not adapted from a previously validated instrument. The items assessed overall happiness (A1), impact of eye disease on happiness (A2), sleep quality (A3), psychological shock at diagnosis (A4), motivation for treatment at diagnosis (A5), perceived change in vision after treatment or disease management (A6), motivation for treatment after initiation (A7), psychological impact of eye disease (A8), quality-adjusted life expectancy (A9), impact of eye disease on other diseases (A10), and impact of other diseases on eye disease (A11).

Each item was scored using an item-specific Likert-type scale, and the score range differed according to the domain. Therefore, each item was analyzed individually rather than aggregated into a single composite score. Participants who were unable to select a valid response category for an individual questionnaire item were treated as having missing data for that item and were excluded only from analyses involving that specific item. For A6, only responses within the predefined 1–5 ordinal scale were considered valid; inability to select a response was treated as non-evaluable rather than as an extreme A6 score. The questionnaire was not intended to diagnose psychological disorders, and no formal psychometric assessment of anxiety, depression, or other psychological states was performed.

### 2.5. Vision-Related Quality of Life

To examine the association between the original questionnaire and an established measure of vision-related quality of life, the 25-item National Eye Institute Visual Function Questionnaire-25 (VFQ-25) was administered [15]. Each item was transformed to a 0–100 scale according to standard scoring procedures, with higher scores indicating better vision-related quality of life. Subscale scores were calculated as the mean of corresponding items. If sub-items were missing, subscale scores were calculated using available responses without imputation, according to standard VFQ-25 scoring procedures. The VFQ-25 composite score was defined as the mean of all vision-targeted subscale scores, excluding the general health item.

### 2.6. Statistical Analysis

All statistical analyses were performed using R-4.6.1 statistical software (R Foundation for Statistical Computing, Vienna, Austria). Continuous variables are presented as the mean ± standard deviation or median with interquartile range, as appropriate. Categorical variables are presented as counts and percentages. All tests were two-sided, and *p* values < 0.05 were considered statistically significant. Because the study was exploratory, all inferential analyses were interpreted as hypothesis-generating.

Associations between age and questionnaire items were evaluated using Pearson or Spearman correlation coefficients, depending on the distribution and measurement level of the variables. To account for multiple comparisons, *p* values were adjusted using the Benjamini–Hochberg procedure to control the false discovery rate. This procedure was applied to the *p* values obtained from the underlying statistical tests; therefore, its applicability depends on the validity of those tests rather than on whether the variables are objectively measured or patient-reported. Participants with non-evaluable responses for a given item were excluded from analyses involving that item only. In particular, participants who were unable to select a valid A6 response category were treated as having missing A6 data and were excluded from all analyses involving A6. Items showing significant associations in the univariate analyses were subsequently evaluated using multivariable linear regression models adjusted for age, disease category, and better-eye logMAR visual acuity. Differences across disease groups were evaluated using the Kruskal–Wallis test. When significant, post hoc comparisons were performed using Dunn’s test with Benjamini–Hochberg correction. Disease-related differences in questionnaire items were further examined using ordinal logistic regression models adjusted for age and better-eye visual acuity. Glaucoma was selected a priori as the reference category because it was the largest disease-specific group and represented a common chronic ophthalmic condition in this cohort. The reference choice was made for presentation and did not imply that glaucoma represented normal vision or a clinically superior comparator. Because perceived visual change after treatment or disease management (A6) showed the strongest association with age in the item-level analyses, its relationships with age and disease category were evaluated in complementary adjusted models using only participants with valid A6 responses. Specifically, the association of A6 with age was assessed in a multivariable model including disease category and better-eye visual acuity, whereas disease-group differences in A6 were assessed using ordinal logistic regression adjusted for age and better-eye visual acuity. Thus, age- and disease-related associations with A6 were evaluated while accounting for the other factors. A formal age-by-disease interaction analysis was not performed because of the exploratory design and the limited sample size in some disease subgroups.

To evaluate robustness, sensitivity analyses were conducted after excluding the visually impaired support group. Associations between selected questionnaire items and the VFQ-25 composite score were assessed using correlation analyses and multivariable linear regression adjusted for age and sex. Internal consistency was assessed using standardized Cronbach’s alpha. ROC analyses were not included in the main analysis because the questionnaire was not intended for diagnostic or screening use.

## 3. Results

### 3.1. Participant Characteristics

A total of 262 participants were included, comprising 88 patients with glaucoma, 51 with AMD, 38 with DMR, 11 with RP, 56 with cataract, and 18 individuals from the visually impaired support group. Significant differences were observed among groups in sex distribution, age, and baseline visual acuity (all *p* < 0.01). The visually impaired group showed markedly worse visual acuity than the disease-specific groups (Table 2). The standardized Cronbach’s alpha of the 11-item questionnaire was 0.74.

### 3.2. Associations Between Age and Questionnaire Items

Several questionnaire items showed negative correlations with age. Perceived visual change (A6) showed the strongest correlation with age (r = −0.271, *p* < 0.001). Significant negative correlations were also observed for happiness impact (A2; r = −0.176, *p* = 0.004), psychological impact (A8; r = −0.151, *p* = 0.014), quality-adjusted longevity (A9; r = −0.132, *p* = 0.032), and initial treatment motivation (A5; r = −0.122, *p* = 0.048). The associations for eye-to-systemic impact (A10; *p* = 0.098) and systemic-to-eye impact (A11; *p* = 0.067) did not reach statistical significance. No significant associations were observed for overall happiness (A1), sleep quality (A3), diagnostic shock (A4), or ongoing treatment motivation (A7). After Benjamini–Hochberg adjustment, only perceived visual change (A6; adjusted *p* < 0.001) and happiness impact (A2; adjusted *p* = 0.023) remained statistically significant.

**Figure 1:**
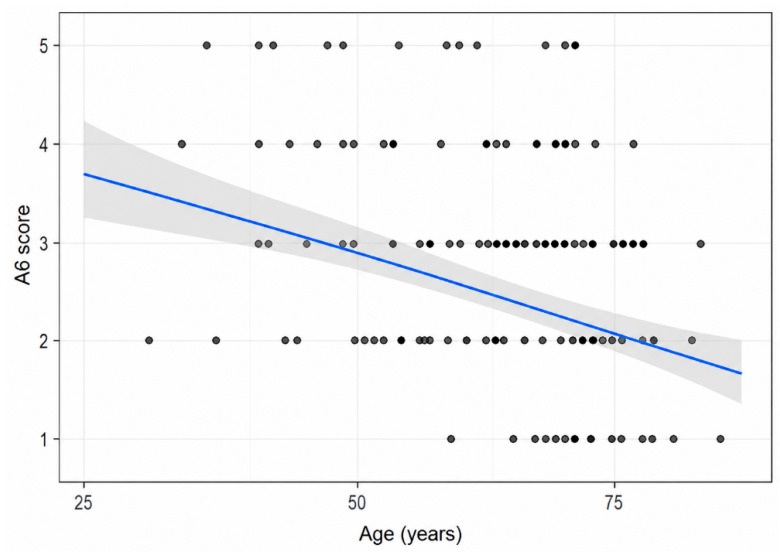
Association between age and perceived visual change after treatment or disease management (A6). The scatter plot shows participants with valid A6 responses within the predefined 1–5 ordinal scale. Participants who were unable to select an A6 response were treated as having missing A6 data and were not included in this figure. The solid line represents the fitted univariable regression line, and the shaded area indicates the 95% confidence interval. This figure is intended for descriptive visualization; inferential interpretation was based on the adjusted multivariable analysis reported in the text.

In multivariable linear regression analyses adjusted for age, disease category, and better-eye visual acuity, the association between happiness impact (A2) and age was no longer statistically significant (β = −0.0033, *p* = 0.475), whereas perceived visual change (A6) remained independently associated with age (β = −0.030, *p* < 0.001). Figure 1 provides a descriptive visualization of the direction, dispersion, and uncertainty of the unadjusted association among participants with valid A6 responses; participants who were unable to select an A6 response were not included in the figure or in A6-specific analyses. Inferential interpretation was based on the adjusted multivariable model. Thus, the age-related association with A6 persisted after adjustment for disease category, while the disease-related differences in A6 described below were estimated after adjustment for age. These complementary analyses indicate that both age and disease category contributed to variation in A6 in this cohort, although no formal age-by-disease interaction was tested.

### 3.3. Association with Visual Acuity

Better-eye visual acuity was not significantly associated with any questionnaire item in either univariate or multivariable analyses.

### 3.4. Disease-Related Differences in Questionnaire Items

In ordinal logistic regression analyses adjusted for age and better-eye visual acuity, several questionnaire items differed across disease groups, with glaucoma as the reference category. After Benjamini–Hochberg correction, 10 comparisons remained statistically significant. For happiness impact (A2), cataract showed lower scores than glaucoma (OR, 0.231; 95% CI, 0.109–0.482; adjusted *p* = 0.001). For sleep quality (A3), RP showed higher scores than glaucoma (OR, 9.61; 95% CI, 2.70–40.4; adjusted *p* = 0.005). For diagnostic shock (A4), cataract showed lower scores than glaucoma (OR, 0.255; 95% CI, 0.127–0.506; adjusted *p* = 0.001).

Among participants with valid A6 responses, for perceived visual change after treatment or disease management (A6), lower scores were observed in AMD (OR, 0.271; 95% CI, 0.141–0.515; adjusted *p* = 0.001), DMR (OR, 0.250; 95% CI, 0.119–0.522; adjusted *p* = 0.002), and cataract (OR, 0.220; 95% CI, 0.109–0.442; adjusted *p* < 0.001), compared with glaucoma. For ongoing treatment motivation (A7), DMR showed lower scores than glaucoma (OR, 0.290; 95% CI, 0.141–0.589; adjusted *p* = 0.004). For psychological impact (A8), cataract showed lower scores than glaucoma (OR, 0.339; 95% CI, 0.171–0.667; adjusted *p* = 0.009). Higher scores were observed in DMR for eye-to-systemic impact (A10; OR, 3.93; 95% CI, 1.84–8.50; adjusted *p* = 0.003) and systemic-to-eye impact (A11; OR, 12.4; 95% CI, 5.55–28.6; adjusted *p* < 0.001), relative to glaucoma. These disease-group differences in A6 were estimated after adjustment for age and better-eye visual acuity, complementing the age-focused multivariable analysis in Section 3.2.

**Figure 2:**
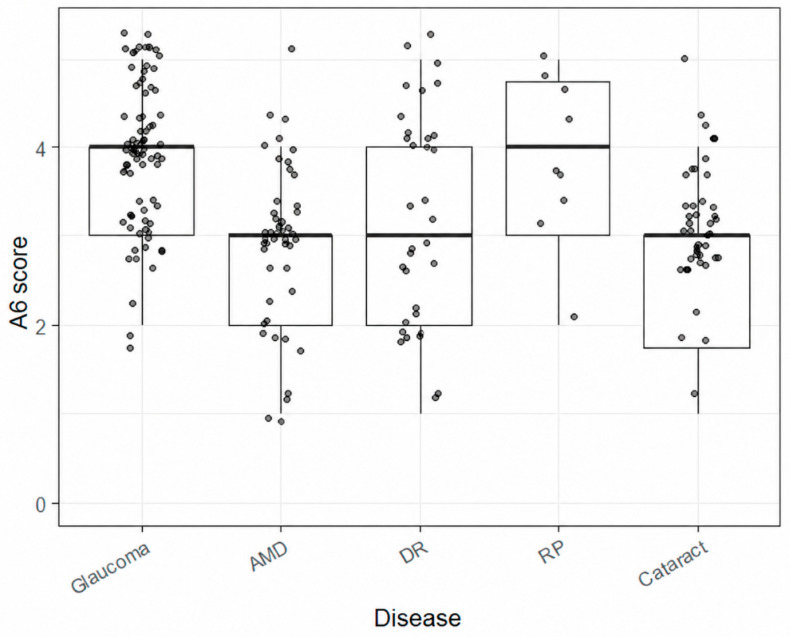
Distribution of perceived visual change after treatment or disease management (A6) across disease groups. Only participants with valid A6 responses within the predefined 1–5 ordinal scale are included; participants who were unable to select an A6 response were treated as having missing A6 data and were excluded. Individual data points are overlaid. Because A6 is an ordinal item, the figure should be interpreted descriptively. For RP, “disease management” may include observation, supportive care, rehabilitation, or counseling rather than a disease-modifying treatment; therefore, A6 should not be interpreted as a direct measure of treatment efficacy.

**Figure 3:**
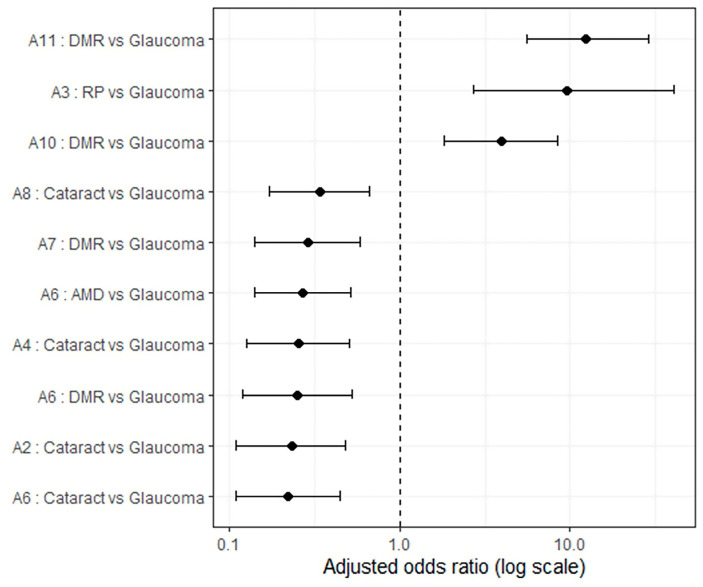
Associations between questionnaire items and disease categories. Forest plot showing adjusted odds ratios from ordinal logistic regression models adjusted for age and better-eye visual acuity, with glaucoma as the reference group. Error bars represent 95% confidence intervals. Abbreviated labels are as follows: happiness impact (A2), sleep quality (A3), diagnostic shock (A4), perceived visual change (A6), ongoing treatment motivation (A7), psychological impact (A8), eye-to-systemic impact (A10), and systemic-to-eye impact (A11). Selection of glaucoma as the reference was for presentation and does not imply normal visual function.

The distribution of perceived visual change after treatment or disease management (A6) by disease group is shown in Figure 2. Adjusted odds ratios for disease-related differences are summarized in Figure 3 using abbreviated item labels together with the A1–A11 identifiers.

### 3.5. Surgical History and Sensitivity Analysis

In univariate analysis, perceived visual change after treatment or disease management (A6) scores were lower in patients with a history of surgery (*p* = 0.024). This association was not significant after adjustment (*p* = 0.61). After excluding the visually impaired support group, A6 remained significantly associated with age and continued to show the strongest association among the questionnaire items.

### 3.6. VFQ-25 Composite Score and Questionnaire Items

**Figure 4:**
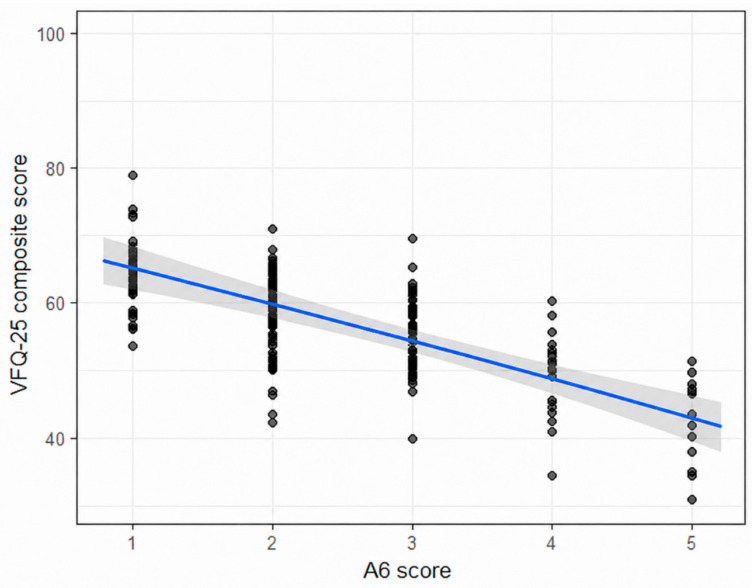
Association between perceived visual change after treatment or disease management (A6) and the VFQ-25 composite score. Only participants with valid A6 responses within the predefined 1–5 ordinal scale are included; non-evaluable A6 responses were treated as missing and excluded. The solid line represents the fitted linear regression line, and the shaded area indicates the 95% confidence interval.

The mean VFQ-25 composite score was 57.9 ± 9.0 (median, 58.3; interquartile range, 52.8–62.2; range, 30.9–100.0). Among participants with valid A6 responses, perceived visual change after treatment or disease management (A6) showed a moderate-to-strong negative correlation with the VFQ-25 composite score (r = −0.693, *p* < 0.001) (Figure 4). In multivariable analysis, A6 remained significantly associated with the VFQ-25 composite score (β = −0.230, *p* < 0.001), whereas age and sex had minimal influence.

## 4. Discussion

### 4.1. Principal Findings

This exploratory cross-sectional study showed that item-level PRO responses differed according to clinical background and were not adequately explained by better-eye visual acuity alone. Among the 11 questionnaire items, perceived visual change (A6) showed the most consistent associations with age, disease category, and VFQ-25 composite score. These findings suggest that A6 may reflect a patient-perceived aspect of visual change related to vision-related quality of life in this cohort, but not necessarily treatment efficacy.

However, these findings should be interpreted cautiously. Important disease-specific clinical variables, including visual field impairment, binocular visual function, contrast sensitivity, structural disease severity, treatment burden, and formal psychological status, were not uniformly available across disease groups. Consequently, A6 should not be interpreted as a comprehensive measure of disease burden, a validated psychological assessment, or an independent clinical indicator.

### 4.2. Item-Level Heterogeneity of Patient-Reported Outcomes

The moderate internal consistency of the questionnaire was consistent with its intended design as a collection of distinct item-level domains rather than a single unified scale. Several items related to psychological impact, treatment motivation, and perceived disease interactions showed disease-specific patterns. Accordingly, we used short descriptive labels together with the A1–A11 identifiers throughout the revised Section 3 and figure legends to facilitate interpretation while preserving correspondence with Table 1.

Previous work has emphasized that PROs in ophthalmology encompass multiple domains, including symptoms, functional limitations, treatment burden, and psychosocial impact [4,8,13,18,19]. The present findings are consistent with this multidimensional concept but should not be considered a formal psychometric validation or structural evaluation of the questionnaire.

Previous studies have identified substantial emotional and mental health burdens associated with eye disease and visual impairment. In an umbrella review, Assi et al. reported that visual impairment, AMD, diabetic retinopathy, and inherited retinal disorders were associated with poorer emotional well-being, depressive symptoms, fear, fatigue, isolation, and impaired psychosocial functioning [9]. Purola et al., using validated instruments including the Beck Depression Inventory and General Health Questionnaire-12, found worse depression and psychological distress scores in several eye-disease groups, although many associations were attenuated after adjustment for age, sex, and comorbidities [10]. Similarly, Gupta et al. and Kandel et al., using the validated multi-item IVI Emotional scale, demonstrated that the emotional impact of eye disease differed according to disease type and severity rather than being uniform across ophthalmic conditions [5,11].

In the present study, sleep quality (A3), diagnostic shock (A4), and psychological impact (A8) did not show consistent associations across the age- and disease-related analyses, although some disease-specific differences were observed. These findings should not be interpreted as evidence that sleep disturbance, depression, or anxiety was absent in this population. Unlike the validated multi-item instruments used in previous studies to assess depression, psychological distress, or vision-specific emotional well-being [5,10,11,20,21], A3, A4, and A8 were single exploratory items. Moreover, the questionnaire did not contain a specific anxiety item and was not designed to diagnose sleep or psychiatric disorders. Patients with known psychiatric disorders were excluded, no control group without ophthalmic disease was included, and many participants had established disease and may have adapted psychologically over time. Heterogeneity in disease severity and treatment burden, together with the limited sample sizes of some subgroups, may also have reduced the ability to detect these effects. Future studies should incorporate validated measures of depression, anxiety, and sleep disturbance, together with disease-specific severity and treatment-burden variables.

### 4.3. Dissociation Between Visual Acuity and Patient Experience

Better-eye visual acuity was not significantly associated with any questionnaire item. This finding is consistent with previous studies showing that visual acuity may correlate only modestly with vision-related quality-of-life measures and may not fully capture what matters to patients in daily life [16,22,23]. Patient priorities may include mobility, reading, face recognition, visual field, contrast sensitivity, binocular function, independence, treatment burden, emotional well-being, and adaptation to visual impairment. Thus, visual acuity is an important outcome but represents only one dimension of patient experience.

### 4.4. Disease-Related Patterns and Confounding

The apparent association between age and A2 disappeared after adjustment for disease category and visual acuity, suggesting that disease composition may partly explain age-related differences in this item. Similarly, the association between surgical history and perceived visual change (A6) was not significant after adjustment, indicating possible confounding by age and clinical background. These findings highlight the importance of careful adjustment when interpreting PROs in observational ophthalmic studies. Participants unable to select a valid A6 response were treated as having missing A6 data rather than as extreme values and were excluded from analyses involving A6. For A6 specifically, age and disease category were examined in complementary mutually adjusted analyses: the association with age remained significant after adjustment for disease category, and disease-group differences were assessed after adjustment for age. This supports the interpretation that both factors may independently contribute to perceived visual change, although the present sample was not designed to test age-by-disease interactions.

Perceived visual change after treatment or disease management (A6) differed across disease categories and was higher in glaucoma than in several other disease groups. This pattern may reflect differences in disease course, treatment expectations, symptom perception, or unmeasured disease-specific clinical variables. Cataract was included as a common and often successfully treatable condition, but its prognosis and treatment pathway differ fundamentally from those of chronic retinal and optic nerve diseases. In RP, disease-modifying treatment is available only for a small, genetically defined subgroup; for most participants, A6 therefore reflects perceived change during disease management, observation, supportive care, rehabilitation, or counseling rather than response to a specific therapy. Accordingly, between-disease differences in A6 should not be interpreted as comparative treatment efficacy.

### 4.5. Clinical Implications

The findings suggest that item-level PRO assessment may provide complementary information when evaluating vision-related quality of life. The results may help treating ophthalmologists identify topics requiring further discussion, help researchers generate hypotheses and select patient-relevant outcomes, and inform collaboration with psychologists or low-vision professionals when appropriate. However, the questionnaire is exploratory and should not be used as a stand-alone instrument for clinical decisions or psychological diagnosis. Longitudinal and disease-specific validation is required before routine clinical application.

### 4.6. Strengths

The strengths of this study include the enrollment of consecutive patients across several major ophthalmic diseases, concurrent administration of an established vision-related quality-of-life instrument, item-level rather than solely composite analysis, adjustment for age and better-eye visual acuity, false-discovery-rate control for multiple comparisons, and sensitivity analysis excluding the heterogeneous visually impaired support group. These features enabled an exploratory comparison of patient perceptions across diverse clinical backgrounds while explicitly preserving the distinction between hypothesis generation and instrument validation.

### 4.7. Limitations

This study has several limitations. First, the cross-sectional design precludes causal inference. Second, the original questionnaire has not been fully validated as an independent instrument, although it showed acceptable internal consistency and an association with the VFQ-25 composite score. Third, formal measures of depression, anxiety, cognitive status, coping, and adaptation were not administered. Consequently, the analyses could not be adjusted for psychological status, and the responses may not fully represent the underlying clinical condition. Exclusion of known psychiatric disorders and dementia reduced, but did not eliminate, this concern. Fourth, important disease-specific variables, including disease severity, visual field impairment, binocular visual function, contrast sensitivity, structural parameters, and treatment burden were not uniformly available. Fifth, the visually impaired support group had heterogeneous underlying diseases and was therefore interpreted descriptively and evaluated in sensitivity analyses. Sixth, coexisting ophthalmic conditions may have influenced responses despite classification by primary diagnosis. Seventh, treatment modalities and prognoses differed substantially across diseases, limiting interpretation of perceived visual change (A6) as a treatment-response measure, particularly in RP. Finally, formal cognitive testing was not performed, and unrecognized mild cognitive decline may have affected responses in this relatively elderly population. In addition, some participants were unable to select a valid response for certain questionnaire items, including A6; these responses were treated as item-specific missing data, which may have reduced the effective sample size for the corresponding analyses.

## 5. Conclusions

Patient-reported outcomes in ophthalmology showed item-level heterogeneity and were not adequately explained by better-eye visual acuity alone. Perceived visual change after treatment or disease management was associated with age, disease category, and VFQ-25 composite score in this exploratory cohort. These findings suggest that patient-perceived visual change may provide complementary, hypothesis-generating information about vision-related quality of life, but the item should not be interpreted as a validated measure of treatment efficacy or psychological status. Further longitudinal studies using validated instruments, formal psychological measures, and disease-specific severity variables are needed to clarify its clinical meaning and applicability.

## Figures and Tables

**Table 1:** Patient-Reported Outcome Items and Scales. Abbreviations: A1–A11 indicate questionnaire items. Higher scores indicate greater levels of the corresponding construct, except where noted. For A2, higher scores indicate a greater negative impact on happiness.

Item	Abbreviated Label	Scale
Overall happiness (A1)	Overall happiness	10 (most)–1 (worst)
Impact of eye disease on happiness (A2)	Happiness impact	4 (worst)–1 (least)
Sleep quality (A3)	Sleep quality	5 (best)–1 (worst)
Psychological shock at diagnosis (A4)	Diagnostic shock	5 (most)–1 (least)
Motivation for treatment at diagnosis (A5)	Initial treatment motivation	5 (most)–1 (least)
Perceived change in vision after treatment or disease management (A6)	Perceived visual change	5 (most)–1 (least)
Motivation for treatment after initiation (A7)	Ongoing treatment motivation	5 (most)–1 (least)
Psychological impact of eye disease (A8)	Psychological impact	5 (most)–1 (least)
Quality-adjusted life expectancy (A9)	Quality-adjusted longevity	5 (≥10 years), 4 (6–9 years), 3 (4–5 years), 2 (1–3 years), 1 (0 years)
Impact of eye disease on other diseases (A10)	Eye-to-systemic impact	5 (most)–1 (least)
Impact of other diseases on eye disease (A11)	Systemic-to-eye impact	5 (most)–1 (least)

**Table 2:** Baseline Characteristics of Participants by Disease Group. *p* values were calculated using the Kruskal–Wallis test for continuous variables and the chi-square test for categorical variables.

Disease	Number	Male (%)	Age, Mean ± SD (95% CI)	Right logMAR	Left logMAR	Better logMAR
				Mean ± SD (95% CI)	Mean ± SD (95% CI)	Mean ± SD (95% CI)
Glaucoma	88	51.1	69.82 ± 11.01 (67.52–72.12)	0.17 ± 0.58 (0.05–0.29)	0.11 ± 0.27 (0.06–0.17)	0.01 ± 0.13 (−0.02–0.03)
AMD	51	68.6	75.78 ± 8.72 (73.39–78.18)	0.18 ± 0.39 (0.07–0.29)	0.20 ± 0.35 (0.10–0.29)	0.01 ± 0.11 (−0.02–0.04)
DMR	38	78.9	63.89 ± 14.35 (59.33–68.46)	0.35 ± 0.49 (0.19–0.50)	0.46 ± 0.59 (0.28–0.65)	0.15 ± 0.31 (0.05–0.25)
RP	11	36.4	57.45 ± 13.15 (49.68–65.22)	0.62 ± 0.68 (0.21–1.02)	0.58 ± 0.71 (0.15–1.00)	0.53 ± 0.71 (0.11–0.95)
Visually impaired group	18	61.1	55.67 ± 17.65 (47.51–63.82)	2.60 ± 0.51 (2.34–2.86)	2.48 ± 0.87 (2.04–2.92)	2.41 ± 0.86 (1.98–2.85)
Cataract	56	53.6	74.86 ± 8.31 (72.69–77.0)	0.35 ± 0.35 (0.25–0.46)	0.37 ± 0.29 (0.29–0.46)	0.37 ± 0.37 (0.28–0.47)
*p* value		<0.001	=0.002	<0.001	<0.001	<0.001

## Data Availability

The datasets generated and/or analyzed during the current study are available from the corresponding author on reasonable request.
